# Validity and reliability of the Neurodevelopmental Screening Tool (NDST) in screening for neurodevelopmental disorders in children living in rural Kenyan coast

**DOI:** 10.12688/wellcomeopenres.16765.1

**Published:** 2021-06-02

**Authors:** Mary A. Bitta, Patricia Kipkemoi, Symon M. Kariuki, Amina Abubakar, Joseph Gona, Jacqueline Philips-Owen, Charles R. Newton

**Affiliations:** 1 Clinical Research-Neurosciences,, KEMRI/Wellcome Trust Research Programme, Centre for Geographic Medicine Research (Coast),, Kilifi, 80108, Kenya; 2 Department of Psychiatry,, University of Oxford, Oxford, OX3 7JX, UK; 3 Department of Public Health,, Pwani University,, Kilifi, 80108, Kenya; 4 Institute of Human Development,, Aga Khan University,, Nairobi,, Kenya; 5 Institute of Psychiatry, Psychology and Neuroscience,, King’s College London, London, UK

**Keywords:** Screening, neurodevelopmental disorders, Africa

## Abstract

**Background: **There are no data on the precise burden of neurodevelopmental disorders (NDD) in Africa, despite high incidence of risk factors. Ten Questions Questionnaire (TQQ) has been used extensively in Africa to screen neurological impairments but not autism spectrum disorders (ASD) and attention-deficit hyperactivity disorders (ADHD). The Neurodevelopmental Screening Tool (NDST) has reliably assessed NDD in Asia; its validity in Africa is unknown.

**Methods: **Using NDST and TQQ, we screened 11,223 children aged 6-9 years in Kilifi, Kenya. We invited all screen-positives and a proportion of screen-negative children for confirmatory diagnosis of NDD using clinical history, neuropsychological assessments and interviews.

**Results: **In total, 2,245 (20%) children screened positive for NDD. Confirmatory testing was completed for 1,564 (69.7%) screen-positive and 598 (6.7%) screen-negative children. NDST’s sensitivity was 87.8% (95%CI: 88.3-88.5%) for any NDD, 96.5% (95%CI:96.1-96.8%) ASD and 89.2% (95%CI: 88.7-89.8%) for ADHD. Moderate/severe neurological impairments’ sensitivities ranged from 85.7% (95%CI: 85.1-86.3%) for hearing impairments to 100.00% (100.0-100.0%) for motor impairments. NDST had higher sensitivities than TQQ for epilepsy (88.8 *vs *86.7), motor impairments (100.0 *vs* 93.7) and cognitive impairment (88.2 *vs* 84.3). Sensitivities for visual and hearing impairments were comparable in both tools. NDST specificity was 82.8% (95%CI: 82.1-83.5%) for any NDD, 94.5% (95%CI: 94.0-94.9%) for ASD and 81.7% (95%CI: 81.0-82.4%) for ADHD. The specificities range for neurological impairments was 80.0% (95%CI: 79.3-80.7%) for visual impairments to 93.8% (95%CI: 93.4-94.3%) for epilepsy. Negative predictive values were generally very high (≤100%), but most positive predictive values (PPV) were low (≤17.8%). Domain specific internal consistency ranged from 0.72 (95%CI: 0.70-0.74) for ADHD to 0.89 (95%CI: 0.87-0.90) for epilepsy.

**Conclusions: **NDST possesses high sensitivity and specificity for detecting different domains of NDD in Kilifi. Low PPV suggest that positive diagnoses should be confirmed when samples are drawn from a population with low disease prevalence.

## Introduction

Neurodevelopmental disorders (NDD) such as autism spectrum disorders (ASD), attention deficit hyperactivity disorders (ADHD) and cognitive impairment are common in low- and middle-income countries (LMIC), but there are few epidemiological studies in Africa ^1^. Despite the high incidence of risk factors for NDD such as environmental toxins, perinatal complications and intracranial infections, the precise burden of these disorders is unknown in Africa ^2^. Few available studies are based on single neurological conditions such as cognitive impairments ^3^, not detecting other important conditions like ASD and ADHD, since the screening was conducted with the Ten Questions Questionnaire (TQQ). The TQQ is a brief and easy to use tool that has been widely used in LMIC to screen for neurological impairment and disability ^4– 6^. This lack of epidemiological data for NDD in Africa is in part ascribed to lack of awareness about their existence in the community, which is now changing following awareness from epidemiological studies of related neurological disorders ^4^. The challenge, however, remains on availability and development of reliable tools for screening and diagnosis of NDD in Africa; this is likely to change given the recent development of a cadre of local psychologists and neurodevelopmental researchers ^7^.

Recently, studies in Asia have developed a tool for screening of developmental disorders in children, specifically the Neurodevelopmental Screening Tool (NDST). This tool was used to screen neurodevelopmental disorders in India, but the estimates differed with region/settings (10–18%) ^8^, which may suggest: (i) that the high burdens of NDD are unique to those regions, and/or (ii) that the reliability and validity of NDST in detecting neurodevelopmental disorders differs across the regions. Although the reliability (test-retest and inter-rater agreement) of NDST was examined and found to be acceptable ^9^, the clinical validity (sensitivities, specificities, positive and negative predictive values) were not reported. It is important to evaluate the validity of screening tools since a tool with low sensitivity results to false-negatives, thereby underestimating the burden of NDD, while those with a low specificity allows false-positives and overestimation of NDD.

The NDST has not been piloted in Africa, yet it allows screening for a wider range of NDD, which have not been assessed together in sub-Saharan Africa and which are often comorbid ^10^. Since the validity and reliability of screening tools may depend on cross-cultural interpretation of the disorders ^6^, it is important to examine the validity of NDST in Africa before its use in the much needed epidemiological studies.

We set up a two-stage large epidemiological study to estimate the burden of neurodevelopmental disorders in a rural area on the Kenyan coast, which allowed us to examine the validity of NDST in detecting NDD in this region. Additionally, we compared the psychometric properties of the NDST to those of the TQQ which has been previously validated for detecting neurological impairments in this setting ^6^. The validity of the TQQ in detecting neurological impairment has been acceptable across different studies, but few studies have compared it with other screening tools for NDD such as NDST. There is need to compare the validity of TQQ with that of NDST in one study.

## Methods

### Ethical statement

Prior to commencement of data collection, ethical approval for this study was obtained from the Scientific and Ethics Review Unit, Kenya under protocol number KEMRI/SERU/CGMR-C/3000. Written informed consent to use and publish de-identified patients’ data was obtained from caregivers of the participants.

### Study settings

This study was conducted in a rural area the of the Kenyan coast in a demographic surveillance area referred to as the Kilifi Health and Demographic Surveillance System (KHDSS) ^11^, which is located in Kilifi County. Vital statistics on births, deaths and migration patterns (in or out) in KHDSS are updated every four months. The main population in KHDSS is the Mijikenda ethnic group, the majority of whom are subsistence farmers and a few fishermen. The literacy levels are low, and Kilifi County is one of the poorest administrative regions in Kenya. The health services for neuropsychiatric and NDD in this area are poorly developed ^12^.

### Sampling

Data were collected between March 2015 and August 2016. The sampling frame for this study comprised children aged 6–9 years living within KHDSS, who form a total population of about 35,000. The age group of 6–9 years was chosen because this is when most NDD become apparent and is when most children in sub-Saharan Africa enrol for school. Each child had an equal probability of being randomly selected to participate in the study so long as they met criteria for age and living within KHDSS. Children would be excluded if they out-migrated or died during the period of study. This validation study was nested in a large epidemiology study which aimed at screening about 15,000 children randomly selected from the 35,000 children. Screening about 15,000 randomly selected children would detect neurodevelopmental disorders with a precision or margin of error of <1%, assuming a conservative prevalence of 6.1% in the community. The RAND () command of MySQL was used to select the eligible children.

### Study design and procedures

The study was implemented using a two-stage design; stage I involved screening of NDD in the community and stage II involved further clinical assessment of all those who screened positive in stage I and a proportion of those who screened negative. Screen- negative participants were included in stage II to obtain reference diagnostic categories for validating against NDST screening status. Eligible participants were identified using the MySQL software then trained interviewers fluent in the local dialects visited the households and explained the study to the parents or close caregivers of eligible children. The NDST and TQQ were then administered to parents who gave informed consent to participate in the study. All children with a positive response in at least one of the NDST items and every 5th child screening negative in all NDST items were invited to the Neuroscience Unit at the KWTRP in Kilifi County Hospital for comprehensive clinical assessments (stage II).

Neurodevelopmental disorders were defined as the presence of either epilepsy, ADHD, ASD, hearing impairments, visual impairments, motor impairments or cognitive impairment. Epilepsy was defined as a history of two or more unprovoked seizures according to recommendations by International League Against Epilepsy (ILAE) ^13^. Hearing impairment was defined as a 41–70 dB loss in the best ear and difficulty in hearing even with a hearing aid. Visual impairment was defined as visual acuity of worse than 6/18. Motor impairment was defined as difficulty in holding implements, dressing and sitting upright but able to move around with help; severe impairment as inability to walk and absence of functional use of hands ^6^. Cognitive impairment was defined as a z *-*score of below −2 in the Ravens Matrix test. Z-scores were calculated as a function of the difference between the mean of an individual child and the mean of a representative sample, divided by the standard deviation of the mean of the representative sample. For standardization, z-scores were calculated separately for each age-year.

Clinical evaluation comprised neuropsychological assessments using structured diagnostic questionnaires as detailed below. A diagnosis of epilepsy was confirmed through clinical history that included presence of seizures as defined by ILAE recommendations ^13^. Cognition was measured using the Ravens Coloured Progressive Matrix Test and Kilifi Naming test ^14^. The Kiddie Schedule for Affective Disorders and Schizophrenia (KSADS ^©^) ^15^ measured ADHD. Autism spectrum disorders diagnosis was done by clinical review using the American Psychiatric Association (APA) Diagnostic and Statistical Manual of Mental Disorders (DSM-V) criteria that followed the Autism Diagnostic Observation Schedule(ADOS ^®^) ^16^ structure. Vision was assessed with Sonksen-Silver Acuity system and hearing assessed with Kamplex screening audiometer model number SM-950.

The questionnaires were administered by trained neuropsychological assessors. All the tools including NDST were translated into the lingua franca, Kiswahili, through a standardised forward and back translation process as in previous studies in the area ^17, 18^. All the tools were piloted to test their comprehensibility and revised accordingly before use in the epidemiological survey. Participants of the pilot tests were randomly selected from community members who attended the Kilifi County Hospital as caregivers of patient. Administration of the tools was supervised by a developmental psychologist, child and adolescent psychiatrist and child neurologist. Final versions of tools used are available as extended data ^19^.

### Statistical analysis

Data were entered using MySQL, and analysed using STATA software (version 13.1, Stata Corp LP, College Station, TX, USA) and R-statistical software (version 3.4.0 (2017-04-21)). Sensitivities, specificities, and positive and negative predictive values of detection of NDD by NDST were computed by comparing screening outcomes in stage I against diagnoses in stage II using the “diagtest” syntax in STATA. Discrete variables were compared using Pearson’s Chi-squared, or Fisher’s exact test where observations in a cell were sparse (less than 5). Internal consistency of the NDST items was assessed by computing the Cronbach’s alpha.

## Results

### General description

**Figure 1.**
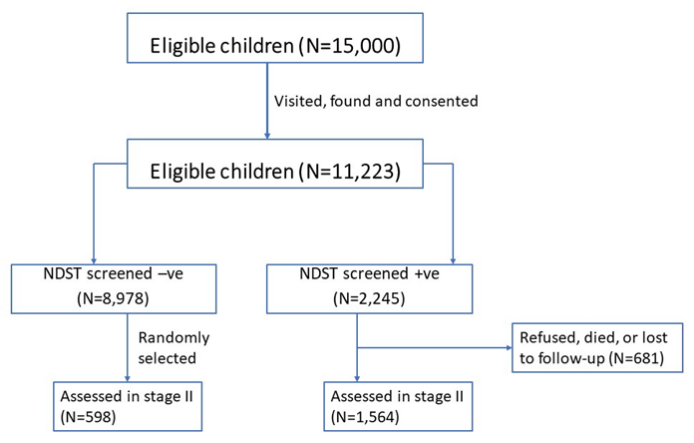
Flow diagram of participant recruitment.

In total, 15,000 children were eligible to participate in the study of whom 11,223 (74.8%) children were available for visitation, consenting and to screening ^19^. The excluded children could not be traced within the KHDSS. Of those who were screened, 49% were female. Of the 11,223 children, 2,245 (20%) screened positive for at least one neurodevelopmental disorder in stage I. In total, 2,162 children were assessed in stage II: 1,564 (69%) of the screen-positive and 598 (6.7%) randomly selected screen negatives. The 681 screen-positive participants from stage I who did not participate in stage II either refused to participate, were lost to follow up or died as summarised in Figure 1. There was no difference in the sexes of those who were assessed in stage II.

### Internal consistency of NDST questions

The total internal consistency of the 39 NDST questions as measured by Cronbach’s alpha was 0.84 (95%CI: 0.82-0.85). The internal consistency of the specific domains ranged from 0.72 (95%CI: 0.70-0.74) for ADHD to 0.89 (95%CI: 0.87-0.90) for epilepsy ( Table 1).

**Table 1.** Internal consistency for the Neurodevelopmental Screening Tool (NDST) questions for each domain measured with Cronbach’s alpha. CI=confidence interval.

Disorder	Number of questions	Cronbach’s alpha (95%CI)
Autism spectrum disorders (ASD)	11	0.82 (0.78-0.86)
Attention deficit hyperactivity disorder (ADHD)	4	0.72 (0.70-0.74)
Epilepsy	2	0.89 (0.87-0.90)
Cognitive impairment	13	0.85 (0.83-0.87)
Motor impairments	4	0.75 (0.69-0.81)
Visual impairments	7	0.74 (0.71-0.77)
Hearing impairments	3	0.76 (0.73-0.79)

### Validity of NDST in detecting all NDD

The false negatives of NDST was 10.1%, with false positive proportion of 17.1%. This translated to a sensitivity of 87.8% (95%CI: 88.3-88.5%) and a specificity of 83.3% (95%CI: 82.7-84.0%). The positive predictive value was low, but the negative predictive value was very high ( Table 2). The sensitivity for having ASD or ADHD (89.5% vs. 88.8%) was higher than for neurological conditions, as was the specificity (82.1% vs. 80.0%) ( Table 2).

**Table 2.** Validity of all Neurodevelopmental Screening Tool (NDST) questions in detecting neurodevelopmental disorders in 11,223 children screened in stage I and 2,162 assessed in stage II.

Impairment	Sensitivity (%) (95% CI)	Specificity (%) (95% CI)	Positive predictive value (%) (95% CI)	Negative predictive value (%) (95% CI)
ASD	96.5 (96.1-96.8)	80.6 (79.9-81.3)	3.7 (3.3-4.00)	99.9 (99.9-100)
ADHD	89.2 (88.7-89.8)	81.7 (81.0-82.4)	11.1 (10.5-11.7)	99.6 (99.5-99.7)
Epilepsy	88.8 (88.2-89.3)	80.6 (79.8-81.3)	3.8 (3.5-4.2)	99.9 (99.8-99.9)
Cognitive impairment	88.2 (87.6-88.5)	80.3 (79.5-81.0)	2.00 (1.7-2.3)	99.9 (99.8-99.9)
Motor impairments	100.0 (100.0-100.0)	80.1 (79.3-80.8)	0.7 (0.6-0.9)	100.0 (100.0-100.0)
Visual impairments	100.0 (100.0-100.0)	80.0 (79.3-80.7)	0.4 (0.3-0.5)	100.0 (100.0-100.0)
Hearing impairments	85.7 (85.1-86.3)	80.0 (79.3-80.7)	0.3 (0.2-0.4)	99.9 (99.9-100.0)
Any neurological impairment*	88.8 (88.3-89.4)	80.0 (79.3-80.7)	0.3 (0.2-0.4)	97.6 (96.9-98.2)
Having ASD or ADHD‡	89.5 (88.9-90.0)	82.2 (81.5-82.9)	13.6 (13.0-14.3)	99.6 (99.5-99.7)
All neurodevelopmental disorders	87.8 (88.3-88.5)	83.3 (82.7-84.0)	20.4 (19.6-21.1)	99.3 (99.1-99.5)

ASD=autism spectrum disorders; ADHD=attention deficit hyperactivity disorder; CI=confidence interval.

### Validity of NDST in detecting presence of ASD or ADHD

ASD was diagnosed in 80 children, of whom 2 (2.5%) were classified as false negatives by NDST in stage I. Of those without ASD in stage II, 2,167/11,143 (19.4%) were classified as false positives by NDST in stage I. Therefore, the sensitivity for NDST in detecting ASD was 96.5% (95%CI: 96.1-96.8%), while the specificity was 80.6% (95%CI: 79.9-81.3%). Screening with ASD specific questions improved the specificity of NDST by about 15% ( Table 3). Positive predictive values of NDST were low but the negative predictive values were high ( Table 2).

**Table 3.** Comparison of validity of all Neurodevelopmental Screening Tool (NDST) questions vs domain specific questions in detecting neurodevelopmental disorders in 11,223 children screened in stage I and 2,162 assessed in stage II.

Impairment	Sensitivity (%) (95% CI)		Specificity (%) (95% CI)		Positive predictive value (%) (95% CI)		Negative predictive value (%) (95% CI)	
	Disorder specific questions	All questions	Disorder specific questions	All questions	Disorder specific questions	All questions	Disorder specific questions	All questions
ASD	94.2 (93.9-96.6)	96.5 (96.1-96.8)	94.5 (94.0-94.9)	80.6 (79.9-81.3)	11.1 (10.5-11.7)	3.7 (3.3-4.00)	99.9 (99.9-100.0)	99.9 (99.9-100)
ADHD	43.2 (42.3-44.1)	89.2 (88.7-89.8)	94.8 (94.4-99.3)	81.7 (81.0-82.4)	17.7 (17.0-18.5)	11.1 (10.5-11.7)	98.4 (98.2-98.7)	99.6 (99.5-99.7)
Epilepsy	61.2 (60.3-62.1)	88.8 (88.2-89.3)	93.8 (93.4-94.3)	80.6 (79.8-81.3)	8.1 (7.6-8.5)	3.8 (3.5-4.2)	36.8 (27.2-46.3)	99.9 (99.8-99.9)

ASD=autism spectrum disorders; ADHD=attention deficit hyperactivity disorder; CI=confidence interval.

ADHD was diagnosed in 280 children, of whom 30 (210.7%) were classified as false negatives by NDST in stage I. Of those without ADHD in stage II, 2,167/11,143 (18.2%) were classified as false positives by NDST in stage I. Therefore, the sensitivity for NDST in detecting ADHD was 89.2% (95%CI: 88.7-89.8%), while the specificity was 81.7% (95%CI: 81.0-82.4%). Screening with ASD specific questions improved the specificity of NDST by 13% ( Table 3). Positive predictive values of NDST were low but the negative predictive values were high ( Table 2).

### Validity of NDST in detecting neurological impairments

False negative classification proportion for epilepsy by NDST was 11/98 (11.2%) while false positives classification was 2,158/11,125 (19.4%). The sensitivity for detecting epilepsy by NDST was 88.8% (88.2-89.3%), with specificity was 80.6% (95%CI: 79.8-81.3%). Use of the question on seizure disorders improved the specificity by 13%. Positive predictive values were low while negative values were high ( Table 2).

False negative classification for cognitive impairment by NDST was 6/51 (11.7%), while false positive classification was 2,200/11,172 (19.6%). The sensitivity for detecting cognitive impairment by NDST was 88.2% (95%CI: 87.6-88.5%), with specificity being 80.3% (95%CI: 79.5-81.0%). Positive predictive and negative values are shown in Table 2.

False negative classification for motor impairments by NDST was 0/16 (0%), while false positive classification was 2229/11207 (19.8%). The sensitivity for detecting moderate/severe motor impairments by NDST was 100.0% (95%CI: 100.0-100.0%), while the specificity was 80.1% (95%CI: 79.3-80.8%). Positive predictive and negative values are shown in Table 2.

False negative classification proportion for visual impairments by NDST was 0/8 (0%), while false positive classification was 2237/11,215 (19.9%). The sensitivity for visual impairments was 100.0% (95%CI: 100.0-100.0%), with specificity being 80.0% (95%CI: 79.3-80.7%). Positive predictive and negative values are shown in Table 2.

False negative classification proportion for hearing impairments by NDST was 1/7 (14.2%), while false positive classification was 2,239/11,216 (19.9%). The sensitivity for detecting hearing impairments by NDST was 85.7% (95%CI: 85.1-86.3%), with specificity being 80.0% (95%CI: 79.3-80.7%). Positive predictive and negative values are shown in Table 2.

### Comparison of NDST with TQQ in detecting neurological disorders

The NDST had higher sensitivities than TQQ for epilepsy, motor impairments and cognitive impairment, but sensitivities for visual and hearing impairments were comparable in both tools. However, TQQ had slightly higher specificities than NDST ( Table 4).

**Table 4.** Validity of all Ten Questions Questionnaire (TQQ) questions in detecting neurological disorders in 11,223 children screened in stage I and 2,162 assessed in stage II. CI=confidence interval.

Impairment	Sensitivity (%) (95% CI)	Specificity (%) (95% CI)	Positive predictive value (%) (95% CI)	Positive predictive value (%) (95% CI)
Cognitive impairment	84.3 (83.6-84.9)	84.4 (83.7-85.0)	2.4 (2.1-2.69)	99.9 (99.8-99.9)
Epilepsy	86.7 (86.1-87.3)	84.7 (79.3-85.3)	4.7 (4.3-5.1)	99.8 (99.7-99.3)
Motor impairments	93.7 (93.3-94.2)	84.2 (83.5-84.8)	0.8 (0.6-1.0)	99.9 (99.9-100.0)
Visual impairments	100.0 (100.0-100.0)	84.1 (83.4-84.8)	0.4 (0.3-0.6)	100.0 (100.0-100.0)
Hearing impairments	85.7 (85.0-86.3)	84.1 (83.4-84.8)	0.3 (0.2-0.4)	99.9 (99.9-100.0)
Any neurological impairment*	86.4 (85.7-87.0)	85.1 (84.4-85.8)	7.8 (7.3-8.3)	99.7 (99.6-99.8)

## Discussion

This study demonstrates that NDST can be reliably used to screen for NDD in rural areas of Kenya. The sensitivities were high for all domains, suggesting that most of the NDD can be identified in the community. The specificities were also high, and therefore little diagnostic resources would be used in confirming a diagnosis of NDD screened with NDST, which is further supported by very high negative predictive values. The study reported low positive predictive values (PPV) which may have been the result of low population prevalence of NDD among randomly sampled children from this community. This low PPV therefore supports the need for setting up a two or three stage study design to confirm diagnosis among screen-positive children. The internal consistency was excellent, underpinning the general relatedness or correlation of all the 39 neurodevelopmental questions.

These results are comparable to those of a study from India ^20^, albeit with some differences. For instance, in the Indian study that tested the tool on 4000 families, NDST achieved optimal sensitivity and specificity using only 11 of the 39 questions ^20^. The differences are not surprising since these studies are from dissimilar settings with unique cultural perspectives that can influence the perception of NDD. Additionally, the present study’s research team had prior experience in conducting neuropsychiatric studies ^4, 21^, which may have improved the ratings of NDST for conditions related to those in previous studies. Lastly, questionnaire adaptation process is crucial and can influence validity and reliability of the tool; focus group discussions and in-depth interviews with community members were applied in Kilifi to understand the local idioms for NDD in this area ^22^.

Not only were sensitivities and specificities of the NDST high in each domain of NDD but were also high in all domains combined, which suggests that the NDST questions can be used to detect the range of NDD. These findings are important in that screening positive in one domain may increase the likelihood of being positive for another domain, as documented in a recent study of epilepsy ^23^, which a few of those who screened negative for seizures in stage I, were found to be positive for epilepsy in stage II. Thus, NDST can be used to reliably measure all NDD in one epidemiological study.

Although sensitivities were high for NDD, it is important to adjust estimates from stage II for sensitivity to avoid underestimating the true burden of the conditions. It is likely that participants not detected by NDST (where sensitivity is not 100%) had mild rather than moderate or severe impairments. Sensitivity is not only determined by the quality of a tool, but also by expertise and training level of staff tasked with identifying NDD in stage II. The good specificities for NDST, especially with use of disorder-specific questions, suggests NDST may be particularly useful in identifying children requiring care in poor regions without the training capacity and diagnostic expertise of NDD. The low positive values advocate for confirmation of the positive status from NDST, which is not a major limitation since most epidemiological studies in Africa often use a two or three stage design to confirm diagnosis ^4, 24^. Given that negative predictive values were very high, only few children screening negative on NDST would need to be evaluated further in stage II, which saves resources particularly for epidemiological studies in low- and middle-income countries.

The slightly higher sensitivities for ADHD and ASD compared to neurological conditions, underlines NDST as the tool of choice for screening ASD and ADHD, for which there are few epidemiological studies in Africa ^12^. The very high sensitivity measures for motor impairments and visual impairments (100%) indicates the ease with which these problems can be detected in the community. For example, moderate to severe motor impairment are easily visible, while moderate to severe visual impairments are detrimental, making them conspicuous and easier to report. Compared to other disorders, hearing impairments had relatively lower sensitivity (85%), which may mean that low concentration/inattention or ear illnesses such as otitis media can be confused for hearing problems in this rural area. Further work on community perception of domains of NDD is justified.

This is the largest epidemiological study to validate the utility of NDST in identifying NDD in low- and middle-income countries. It is robust in that focus was given to many domains of NDD. One limitation is the lack of detailed assessment of cognitive impairment that may results in lower sensitivities and specificities of NDST. Neuropsychological assessments can be particularly intensive when administered in over 2,000 participants as in this study, and so NDST could be further evaluated in smaller case-control studies in which more tests of cognitive impairment are feasible.

The NDST appeared superior compared to the TQQ. Firstly, NDST can screen for ASD and ADHD in addition to neurological conditions, while TQQ focuses mostly on the latter. Although TQQ’s sensitivity for visual and hearing impairments compared well with that of NDST, the lower sensitivities for epilepsy, motor impairment and cognitive impairment may result in the underestimation of these conditions and NDD in general. Slightly higher specificities for TQQ over NDST would not add much value, since the relative strength of screening tools should be their ability to detect all conditions during screening, which is determined by high sensitivities rather than specificities. These two conclusions taken together with the fact that TQQ does not ask specific questions for ASD and ADHD places NDST as the screening tool of choice for all NDD.

In conclusion, this study shows that NDST can be used reliably to screen different types of NDD in a rural region of Africa, with high sensitivity and specificity. The tool could reduce the cost of identifying children requiring management for NDD in similar settings in Africa or elsewhere. Low positive predictive values suggest that the prevalence of NDD may be low in this community therefore a multi-stage epidemiological study design is recommended, so that diagnosis can be confirmed at later stages of studies by trained staff, clinicians or experts.

## Data availability

### Underlying data

Harvard Dataverse: Replication Data for: Validity and reliability of the Neurodevelopmental Screening Tool (NDST) in screening for neurodevelopmental disorders in children living in rural Kenyan coast. https://doi.org/10.7910/DVN/Z847EG ^19^.

This project contains the following underlying data:

- NDST KILIFI DATASET 23MARCH2021-1.tab (data used in calculating the validity of the NDST in assessing neurodevelopmental disorders in a community sample of 6–9-year-old children from Kilifi, Kenya)
- Some NDST paper codes21March2021.do (STATA v15.1 analysis script)

### Extended data

Harvard Dataverse: Assessment tools and participant materials for: Validity and reliability of the Neurodevelopmental Screening Tool (NDST) in screening for neurodevelopmental disorders in children living in rural Kenyan coast. https://doi.org/10.7910/DVN/Z847EG ^19^.

This project contains the following extended data:

- MBitta_NDST_Kilifi_Codebook.pdf (Variable codebook containing description, value labels and format - English Version)
- MBitta_NDST_Kilifi_Readme.txt (Readme file containing information on the related research study, terms of access, citation requirements as well as methods of processing)
- NDD 2015 - NDST-English.doc (The English version of the Neurodevelopmental Screen Questionnaire that was used to screen participants at stage I of the study)
- NDD 2015 - NDST-Kigiriama.doc (The Giriama version of the Neurodevelopmental Screen Questionnaire that was used to screen participants at stage I of the study)
- NDD 2015 - NDST-Kiswahili.doc (The Kiswahili version of the Neurodevelopmental Screen Questionnaire that was used to screen participants at stage I of the study)
- NDD 2015 – NeuroExamination (The English version of the clinical evaluation form used to collect data on confirmed diagnoses at stage II)
- NDD 2015 - TQQ.doc (The English version of the Ten Questions Questionnaire that was used to screen participants at stage I of the study)
- Socio demo.docx ((The English version of the Sociodemographic Questionnaire)
- Interview ICF_NDD study.doc (The English version of the Informed Consent form)

Data are available under the terms of the Creative Commons Attribution 4.0 International license (CC-BY 4.0).

## References

[1] Bitta M Kariuki SM Abubakar A : Burden of neurodevelopmental disorders in low and middle-income countries: A systematic review and meta-analysis [version 3; peer review: 2 approved, 2 approved with reservations]. * Wellcome Open Res.* 2017;2:121. 10.12688/wellcomeopenres.13540.3 29881784 PMC5964629

[2] Newton CR : Neurodevelopmental disorders in low- and middle-income countries. *Dev Med Child Neurol.* 2012;54(12):1072. 10.1111/j.1469-8749.2012.04384.x 22803812

[3] Durkin MS Hasan ZM Hasan KZ : Prevalence and correlates of mental retardation among children in Karachi, Pakistan. *Am J Epidemiol.* 1998;147(3):281–8. 10.1093/oxfordjournals.aje.a009448 9482503

[4] Mung’ala-Odera V Meehan R Njuguna P : Prevalence and risk factors of neurological disability and impairment in children living in rural Kenya. *Int J Epidemiol.* 2006;35(3):683–8. 10.1093/ije/dyl023 16492712

[5] Durkin MS Davidson LL Desai P : Validity of the ten questions screened for childhood disability: results from population-based studies in Bangladesh, Jamaica, and Pakistan. *Epidemiology.* 1994;5(3):283–9. 7518697

[6] Mung’ala-Odera V Meehan R Njuguna P : Validity and Reliability of the ‘Ten Questions’ Questionnaire for Detecting Moderate to Severe Neurological Impairment in Children Aged 6–9 Years in Rural Kenya. *Neuroepidemiology.* 2004;23(1–2):67–72. 10.1159/000073977 14739570

[7] Bojuwoye O : Training of Professional Psychologists for Africa: Community Psychology or Community Work? *J Psychol Afr.* 2006;16(2):161–6. 10.1080/14330237.2006.10820117

[8] Silberberg D Arora N Bhutani V : Neuro-Developmental Disorders in India - From Epidemiology to Public Policy (I10-1.006). NS Poster Session 2014: Neurology;2014. Reference Source

[9] Silberberg D Arora N Bhutani V : Neuro-Developmental Disorders in India – An INCLEN Study (P04.229).P04 Global Health; 2013: Neurology;2013.

[10] Mpaka DM E-Andjafono Okitundu DL Ndjukendi AO : Prevalence and comorbidities of autism among children referred to the outpatient clinics for neurodevelopmental disorders. *Pan Afr Med J.* 2016;25:82. 10.11604/pamj.2016.25.82.4151 28292045 PMC5324163

[11] Scott JA Bauni E Moisi JC : Profile: The Kilifi Health and Demographic Surveillance System (KHDSS). *Int J Epidemiol.* 2012;41(3):650–7. 10.1093/ije/dys062 22544844 PMC3396317

[12] Bitta MA Kariuki SM Chengo E : An overview of mental health care system in Kilifi, Kenya: results from an initial assessment using the World Health Organization’s Assessment Instrument for Mental Health Systems. *Int J Ment Health Syst.* 2017;11:28. 10.1186/s13033-017-0135-5 28416966 PMC5392905

[13] Fisher RS Acevedo C Arzimanoglou A : ILAE official report: a practical clinical definition of epilepsy. *Epilepsia.* 2014;55(4):475–82. 10.1111/epi.12550 24730690

[14] Kitsao-Wekulo P Holding PA Kvalsvig JD : Measurement of expressive vocabulary in school-age children: Development and application of the Kilifi Naming Test (KNT). *Appl Neuropsychol Child.* 2019;8(1):24–39. 10.1080/21622965.2017.1378579 29023138 PMC6474720

[15] Kaufman J Birmaher B Brent D : Schedule for Affective Disorders and Schizophrenia for School-Age Children-Present and Lifetime Version (K-SADS-PL): initial reliability and validity data. *J Am Acad Child Adolesc Psychiatry.* 1997;36(7):980–8. 10.1097/00004583-199707000-00021 9204677

[16] Lord C Rutter M Goode S : Autism diagnostic observation schedule: a standardized observation of communicative and social behavior. *J Autism Dev Disord.* 1989;19(2):185–212. 10.1007/BF02211841 2745388

[17] Abubakar A Kalu RB Katana K : Adaptation and Latent Structure of the Swahili Version of Beck Depression Inventory-II in a Low Literacy Population in the Context of HIV. *PLoS One.* 2016;11(6):e0151030. 10.1371/journal.pone.0151030 27258530 PMC4892521

[18] Kariuki SM Abubakar A Murray E : Evaluation of psychometric properties and factorial structure of the pre-school child behaviour checklist at the Kenyan Coast. *Child Adolesc Psychiatry Ment Health.* 2016;10:1. 10.1186/s13034-015-0089-9 26793272 PMC4719674

[19] Bitta M Kipkemoi P Kariuki S : Replication Data for: Validity and reliability of the Neurodevelopmental Screening Tool (NDST) in screening for neurodevelopmental disorders in children living in rural Kenyan coast.Harvard Dataverse, V4, UNF: 6:Gqn1nvynujOJ+9/XGoeQRA== [fileUNF].2021. 10.7910/DVN/Z847EG PMC8503789 34676305

[20] Silberberg D : Neurodevelopmental Disorders in India: From Epidemiology to Public Policy.2014; (accessed 11/03 2021). Reference Source

[21] Kariuki SM Abubakar A Kombe M : Burden, risk factors, and comorbidities of behavioural and emotional problems in Kenyan children: a population-based study. *Lancet Psychiatry.* 2017;4(2):136–45. 10.1016/S2215-0366(16)30403-5 28137381 PMC5285446

[22] Abubakar A Kariuki SM Tumaini JD : Community perceptions of developmental and behavioral problems experienced by children living with epilepsy on the Kenyan coast: A qualitative study. *Epilepsy Behav.* 2015;45:74–8. 10.1016/j.yebeh.2015.02.023 25868003 PMC5257264

[23] Kind CJ Newton CRJC Kariuki SM : Prevalence, risk factors, and neurobehavioral comorbidities of epilepsy in Kenyan children. *Epilepsia Open.* 2017;2(4):388–99. 10.1002/epi4.12069 29588970 PMC5862110

[24] Ngugi AK Bottomley C Kleinschmidt I : Prevalence of active convulsive epilepsy in sub-Saharan Africa and associated risk factors: cross-sectional and case–control studies. *Lancet Neurol.* 2013;12(3):253–63. 10.1016/S1474-4422(13)70003-6 23375964 PMC3581814

