# Supplementary material for: Validity and reliability of the Neurodevelopmental Screening Tool (NDST) in screening for neurodevelopmental disorders in children living in rural Kenyan coast
Source: Wellcome Open Res. 2021 Jun 2;6:137. [Version 1] doi: 10.12688/wellcomeopenres.16765.1 (PMC8503789; doi:10.12688/wellcomeopenres.16765.1)
Supplement: Peer review history

# Peer review: Reviewer response for version 1

**DOI:** 10.21956/wellcomeopenres.18490.r45939

**Published:** 2021-10-08

**Authors:** Jean-Baptiste Le Pichon

**Affiliations:** 1 Department of Pediatrics, Children's Mercy Hospital,, University of Missouri-Kansas City School of Medicine, Kansas City, MO, USA

In the paper “Validity and reliability of the Neurodevelopmental Screening Tool (NDST) in screening for neurodevelopmental disorders in children living in rural Kenyan coast” Bitta *et al *present a comparative prospective cohort epidemiological study comparing the efficacy of the Neurodevelopmental Screening Tool (NDST) to the Ten Question Questionnaire (TQQ) in assessing neurodevelopmental disabilities (NDD). This study was conducted in Kifili County in Kenya. The test population was randomly sampled from 30,000 children ages 6 to 9 years old. A total of 15,000 children were tested, of those approximately 11,200 qualified for the study. All of the patients who screened positive for at least one item on the NDST and 20% of the screen negative patients were further evaluated with formal neuropsychological testing. In the end the authors find that approximately 20% of the patients screened positive for neurodevelopmental disabilities by either the NDST or the TQQ. Of those, 69% were available for further neuropsychological testing. The NDST test was found to have a specificity of 83% and a sensitivity of 88%. Remarkably the negative predictive values of the tests in detecting NDD and other neurological impairments (ADHD, ASD, etc.) approached 100% in all cases. The positive predictive value ranged from less than 1% to 20%. The NDST and the TQQ were very comparable in their sensitivity and specificity for all the variables measured. The benefit of the NDST being that it allows screening for ADHD and ASD.

 Strength: This is a well conducted study on a large sample of patients. The authors were careful in ensuring that randomness was maintained in as much as possible, minimizing the risk of sampling error. The extent to which they went to confirm the diagnoses of ADHD, autism, and other neurodevelopmental disorders is quite remarkable, including among other tests the Kiddie Schedule for Affective Disorders and Schizophrenia and the Autism Diagnostic Observation Schedule.

 Conclusion:

 This is an important epidemiological study on the incidence of neurodevelopmental disorders conducted in a sub-Saharan population. The study is important for several reasons:

1. It validates the NDST tool as a measure of NDD, including ADHD and autism in a sub-Saharan context.
2. It is a proof of concept that such epidemiological studies can and should be conducted in sub-Saharan Africa.
3. It sets up the stage for further studies targeted to children with NDDs, allowing targeting to subgroups, including ADHD and autism.

This is a monumental effort in a sub-Saharan country proving that excellent epidemiological research can be done in low- and middle-income countries and preparing the terrain for further studies in low and middle income countries for children with disabilities that have been largely neglected until now.

 Questions:

1. How was the patient population selected? The authors state that the patients were randomly selected from 30,000 eligible children. But it is not clear if this represents the entire population of eligible children in the Kilifi Heath and Demographic Surveillance System (Kilifi County has a population of about 1.5 million, I am not sure I understand how much of this population is served by the Kilifi Health and Demographic Surveillance System).
2. It is my understanding that the Mijikenda ethnic group the patients were sampled from speak a variety of dialects related to Swahili. The tests were translated in Kiswahili. Is Kishwahili the common language for all the Mijikenda people, or were some populations excluded if they could not understand Kiswahili?
3. One of the cognitive tests used to evaluated intellectual disability was Raven’s Progressive Matrices. Yet, it is not clear that this test has been established as a valid measure of IQ in Sub-Saharan Africa. In fact, at least one study suggests otherwise (Wicherts, Jelte M., *et al*. "Raven's test performance of sub-Saharan Africans: Average performance, psychometric properties, and the Flynn Effect." *Learning and Individual Differences* 20.3 (2010): 135-151.). It would be helpful to have the authors comment on this issue.

Suggestions:

- It would be useful to have the results for the NDST and TQQ shown in the same table or in the form of a bar graph. This would make direct comparison of the tests visually much easier.

Minor points:

- The abbreviation KWTRP is never defined.

Is the work clearly and accurately presented and does it cite the current literature?

Yes

If applicable, is the statistical analysis and its interpretation appropriate?

Yes

Are all the source data underlying the results available to ensure full reproducibility?

Yes

Is the study design appropriate and is the work technically sound?

Yes

Are the conclusions drawn adequately supported by the results?

Yes

Are sufficient details of methods and analysis provided to allow replication by others?

Yes

Reviewer Expertise:

Child Neurology and epilepsy

I confirm that I have read this submission and believe that I have an appropriate level of expertise to confirm that it is of an acceptable scientific standard.

## References

[1] : Raven's test performance of sub-Saharan Africans: Average performance, psychometric properties, and the Flynn Effect. *Learning and Individual Differences* .2010;20(3) : 10.1016/j.lindif.2009.12.001 135-151 10.1016/j.lindif.2009.12.001

# Peer review: Reviewer response for version 1

**DOI:** 10.21956/wellcomeopenres.18490.r45941

**Published:** 2021-09-27

**Authors:** Manoja Kumar Das

**Affiliations:** 1 The INCLEN Trust International, New Delhi, Delhi, India

1. Methodology: Whether NDST and TQQ interviews were blinded (i.e. same person interviewed both NDST and TQQ or different persons? If the same person conducted both interviews, what was the interval between two interviews?).
2. Typo error on page five (second line): Screening with ADHD-specific question, instead of ASD question).
3. I think the NDST tool used in this study was developed by the INCLEN team. The source of the NDST tool should be mentioned in the text. The authors should acknowledge INCLEN in the acknowledgment section.  
4. In the discussion, the study on NDD from India should be mentioned (Arora *et al.*, 2018 ^1^).

Is the work clearly and accurately presented and does it cite the current literature?

Partly

If applicable, is the statistical analysis and its interpretation appropriate?

Yes

Are all the source data underlying the results available to ensure full reproducibility?

Yes

Is the study design appropriate and is the work technically sound?

Yes

Are the conclusions drawn adequately supported by the results?

Yes

Are sufficient details of methods and analysis provided to allow replication by others?

Yes

Reviewer Expertise:

Child health, child development

I confirm that I have read this submission and believe that I have an appropriate level of expertise to confirm that it is of an acceptable scientific standard.

## References

[1] : Neurodevelopmental disorders in children aged 2-9 years: Population-based burden estimates across five regions in India. *PLoS Med* .15(7) : 10.1371/journal.pmed.1002615 e1002615 10.1371/journal.pmed.1002615 30040859PMC6057634
